# Two-dimensional Infrared Spectroscopy Reveals Better Insights of Structure and Dynamics of Protein

**DOI:** 10.3390/molecules26226893

**Published:** 2021-11-16

**Authors:** Kiran Sankar Maiti

**Affiliations:** 1 Max-Planck-Institut für Quantenoptik, Hans-Kopfermann-Straße 1, 85748 Garching, Germany; kiran.maiti@mpq.mpg.de; Tel.: +49-89-289-54056; 2 Lehrstuhl für Experimental Physik, Ludwig-Maximilians-Universität München, Am Coulombwall 1, 85748 Garching, Germany

**Keywords:** vibrational spectroscopy, VSCF, pair potential, one-dimensional IR, two-dimensional IR, dual frequency, hydrogen bond, amide bond, vibrational coupling

## Abstract

Proteins play an important role in biological and biochemical processes taking place in the living system. To uncover these fundamental processes of the living system, it is an absolutely necessary task to understand the structure and dynamics of the protein. Vibrational spectroscopy is an established tool to explore protein structure and dynamics. In particular, two-dimensional infrared (2DIR) spectroscopy has already proven its versatility to explore the protein structure and its ultrafast dynamics, and it has essentially unprecedented time resolutions to observe the vibrational dynamics of the protein. Providing several examples from our theoretical and experimental efforts, it is established here that two-dimensional vibrational spectroscopy provides exceptionally more information than one-dimensional vibrational spectroscopy. The structural information of the protein is encoded in the position, shape, and strength of the peak in 2DIR spectra. The time evolution of the 2DIR spectra allows for the visualisation of molecular motions.

## 1. Introduction

Proteins are the building block of the biological system and perform most of the chemical and biological processes of the living body [1,2,3]. During the chemical and biological processes, proteins are constantly in motion and undergo conformational change [4,5,6]. To understand the biological processes, it is absolutely necessary to know the structure and the dynamics of the protein [7,8]. There are many theoretical and experimental techniques available to predict or visualise the three-dimensional structure and vibrational motion of the proteins [9], some of which are nuclear magnetic resonance (NMR) spectroscopy [10,11,12], X-ray crystallography [13,14,15], fluorescence spectroscopy [2], vibrational spectroscopy [16,17,18], and so forth. Unfortunately, proteins are large in size and extremely complex in structure. In addition, the dynamical processes of the protein take place in a large range of timescales, from femtoseconds (fs) to milliseconds (ms) [19]. Due to the extreme complexity of the protein structure and dynamics, none of the above-mentioned methods are self-sufficient. For example, very accurate structural information is possible to get using X-ray crystallography techniques; however, dynamical information is lost from this technique. Moreover, due to the freezing of the molecule, the structure from crystallography is different from the real structure in the condensed phase [9]. NMR spectroscopy, especially multidimensional NMR spectroscopy, is able to provide the three-dimensional structure as well as the dynamical information of the protein conformational change [12]; however, NMR resolves the slow processes only in the range of μs [20]. Therefore, the fast conformational changes of proteins (typically in the “fs” time scale) remain obscured with NMR spectroscopy. Vibrational spectroscopy, especially after the development of an ultrafast laser system, manifests a great promise to reveal the structure and fast molecular motion of the proteins [21,22]. In principle, the dynamical information content of vibrational spectroscopy is analogous to NMR spectroscopy; however, it provides information much faster in time-scales and practically probes the molecular vibration in real time [23,24].

In vibrational spectroscopy, infrared radiation is being used to excite the vibrational modes of the molecule of interest [16,25]. Typically, the mid-infrared (MIR) light source (spectral range from 2.5 to 20 μm) is used to excite the fundamental vibrational and rotational bands of the molecule [26,27]. These vibrational resonances are used as a probe to reveal the structure and vibrational dynamics of the molecule [28,29,30,31]. The conventional vibrational spectroscopy technique provides a one-dimensional (1D) projection of the available molecular information of a sample onto a single frequency axis. A typical one-dimensional vibrational spectrum of protein often contains several amide bands with vibrational contributions from both protein backbone, and amino acid side chains. In particular, amide **I**, which is associated with C=O stretch vibration and amide **A**, associated with N-H stretch vibrations are more important in protein research [32,33,34]. Since both C=O and N—H vibrational bonds are involved in the hydrogen bonding between different moieties of secondary structure, the positions of both amide **I** and amide **A** bands are sensitive to the secondary structure composition of a protein. Unfortunately, 1D spectroscopy is unable to provide information on the amide **I** and amide **A** interaction. In contrast, multidimensional spectroscopy techniques provide a multidimensional projection of the relevant molecular motions offering enormously more information than 1D spectroscopy. Due to the multidimensional projection of molecular motions, coupling information is readily available from multidimensional spectra [23].

One of the most successful ultrafast multidimensional vibrational spectroscopic approaches is two-dimensional infrared (2DIR) spectroscopy [35]. In 2DIR spectroscopy, the coherent higher-order polarization of the sample induced by the sequence of infrared pulses is projected in two-dimensional (2D) frequency space [23,36]. The structural, as well as dynamical information, are encoded in the position, shape, and strength of the two-dimensional peaks (similarly as 2D NMR spectra) and their respective temporal evolution [37]. The interpretation of the 2D data in terms of a dynamical model of the protein under inspection requires substantial theoretical modeling. In this review article, it has been shown how a multidimensional approach provides a better insight into molecular information. The discussion will start with the theoretical approach, and show how multidimensional potential energy surfaces improve the anharmonic vibrational frequency calculations and continue with the experimental 2DIR approach to get structural and dynamical information of the molecules.

## 2. Vibrational Spectra of Protein

Vibrational absorption spectra of proteins and peptides are influenced by vibrational bands that can be expressed approximately in terms of oscillators localized in each repetitive unit and their mutual couplings [38,39]. Among all the vibrational bands of protein and peptides, amide **A** and amide **B** in the spectral window between 3000–3500 cm${}^{-1}$ and amide **I** and amide **II** between 1500 and 1700 cm${}^{-1}$ are the most extensively studied vibrational bands for researchers [7,19,40]. The reasons for the selection of particularly amide bands are that firstly, these vibrational bands are spectrally well-separated from the remaining spectrum of protein, and secondly, amide bands reveal a robust dependence on the structural motifs present in the investigated peptides and proteins. The amide **I** vibrational mode, which is practically the C=O stretch vibration, has experimentally, as well as theoretically been the most important and well-studied vibrational mode due to its large transition-dipole moment. In addition, it seems to be a mostly decoupled mode from the rest of the vibrational modes in peptides and proteins. There may be a few hundreds of theoretical as well as experimental articles dealing with amide **I** bands [18,24]. However, with the development of computational power and ultrafast pulsed laser, C-H stretch vibrations, coupling of the C-H vibrational mode with amide bonds, hydrogen bonding, coupling of different amide bonds, and so forth have gradually become important in the vibrational spectroscopy of proteins. An extensive analysis of the above-mentioned vibrational bands is therefore mandatory to reveal the structure and dynamics of the peptide and protein. Vibrational analysis of those bands even for the smallest protein (containing about 50 amino acid residues [41]) is extremely difficult theoretically as well as experimentally. As a model, N-Methylacetamide, commonly known as NMA, containing a single amino acid unit, is well-studied theoretically and experimentally to understand the protein structure and dynamics. In this review, I will show the above-mentioned structural information by analyzing two model molecules, Methyl benzoate (MB) and 2-Pyrolidinone, where they have similar structural bands as proteins.

## 3. Theoretical Approach

The vibrational frequency calculation within the harmonic approximation is advantageous; however, it generally has insufficient significance because most of the biologically relevant molecules are ‘‘floppy’’ and influenced by strong anharmonic effects [34,42]. Strong anharmonic effects are observed particularly in weakly bound molecular complexes, for example, hydrogen-bonded complexes with surrounding water [43,44,45,46,47]. Moreover, in molecular dynamics, it is more interesting to study molecular configurations that are far from the equilibrium, where the harmonic approximation is unrealistic. The foremost difficulty of anharmonic vibrational frequency calculations for large molecular systems is that the vibrational bonds are not mutually separable as in the harmonic approximation [38]. Therefore, it is necessary to calculate the energy levels and wavefunctions for the systems of many coupled degrees of freedom. A large number of studies have been carried out to overcome this problem. Among many, diffusion quantum Monte Carlo (DQMC) [48,49,50], the discrete variable representation (DVR) [51,52,53], vibrational self-consistent field (VSCF) [54,55,56,57], and vibrational configuration interaction (CI) [58,59,60] methods manifested their applicability to calculate anharmonic effects in molecular systems with different sizes. In the case of large molecular systems, the VSCF method is the most successful among the above-mentioned methods to effectively calculate vibrational spectra.

### 3.1. VSCF Method

The success of the VSCF method relies on the accuracy of the calculated potential energy surfaces (PES) accounting for possible coupling effects. The PES in the VSCF expansion can be expressed in terms of a hierarchical expansion

(1) $$\begin{array}{ccc} V(q_{1},\cdots ,q_{N}) & = & \sum_{j}^{N}V_{j}^{\left(1\right)}\left(q_{j}\right)+\sum_{i<j}V_{i,j}^{\left(2\right)}\left(q_{i},q_{j}\right)+\sum_{i<j<k}V_{i,j,k}^{\left(3\right)}\left(q_{i},q_{j},q_{k}\right)+\cdots \\ & + & \sum_{i<j\cdots <r<s}V_{i,j,\cdots ,r,s}^{\left(n\right)}\left(q_{i},q_{j},\cdots ,q_{r},q_{s}\right)+\cdots . \end{array}$$

where $V_{j}^{\left(1\right)}\left(q_{j}\right)$ are the one-dimensional potentials, $V_{i,j}^{\left(2\right)}\left(q_{i},q_{j}\right)$ are the pairwise coupling potentials, $V_{i,j,k}^{\left(3\right)}\left(q_{i},q_{j},q_{k}\right)$ are the triple coupling potentials, and so on. The calculation of the PES is one of the most tedious parts of VSCF calculation, especially when pairwise and higher coupling potentials are calculated. A detailed analysis of the coupling effect has been documented in the Refs. [61,62].

**Figure 1:**
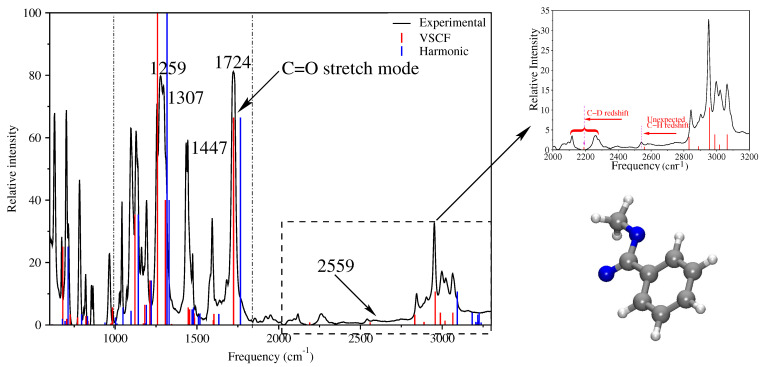
Experimental one-dimensional infrared (1DIR) spectra (black line) of deuterated MB. The calculated harmonic (blue sticks) and anharmonic (red sticks) vibrational frequencies are plotted along with experimental spectra [62]. The intensity of the calculated harmonic frequencies are also used to plot anharmonic frequencies.

The VSCF method has been tested by several researchers and established as a reliable technique for the anharmonic vibrational frequency calculation of biological molecules [38,62,63,64]. It is used here to compute the anharmonic vibrational frequencies of MB and demonstrates how pairwise (two-dimensional) and higher-order couplings (multidimensional) improve the anharmonic frequency calculations [61]. MB is selected as a model molecule to demonstrate the vibrational couplings among the carbonyl group and a side-chain C–H moiety. This coupling information would allow for the determination of backbone and side chain dynamics in peptides and proteins. In addition, the C=O double-bond structure in the carboxylic ester group of MB necessarily represents a local oscillator like the amide **I** band in proteins and provides a convenient mode for the model studies [65,66,67]. Diagonal PES has been calculated along with pairwise, as well as triple coupling potentials. Ideally, closely spaced potential points are desirable for a better description of PES. Unfortunately, computational cost increases very fast with the number of potential points, and particularly in the case of pairwise and triple coupling potentials, computational cost increases exponentially. To keep the computational cost reasonable, it is a common practice to calculate a few unevenly spaced potential points in the PES and finally extrapolate them to get evenly spaced denser potential points in the PES [62]. In addition to that, basis set extrapolation is used to generate highly accurate PES with much lower computational cost [68]. All the potential points have been calculated using different computational methods and varying sizes of basis sets [62,69]. A significant improvement has been observed when pair-coupling PESs has been used to calculate an anharmonic frequency. The calculated harmonic and anharmonic frequencies are plotted along with the experimental spectra in Figure 1.

The calculated anharmonic frequencies using the VSCF method have been compared to the experimental frequencies [65]. The root mean squared deviation (RMSD) is 151 for the anharmonic frequencies calculated using only 1D potential. A significant improvement was observed in frequency calculation using pairwise PESs and the calculated RMSD is 24 only. The calculation of the pairwise potential mean considering the coupling of two vibrational bands, it practically symbolized the two-dimensional PES. Since the frequency calculation was improved significantly by using 2D PES, it was therefore expected that triple and higher-order terms in the many-body expansion of PESs should have a notable impact on the anharmonic vibrational frequency calculation. For a few selected difficult modes, the PM3 triple coupling potentials have been considered in the calculation; however, improvement was rather insignificant and not at all price-worthy. Therefore, it can be inferred that two-dimensional PES is good enough to reveal the molecular detail for moderate-size molecular systems [70].

### 3.2. C-H Vibrational Bands

As mentioned before, the majority of research on vibrational spectroscopy of protein deals with the different amide bands. The rest of the vibrational bands are so far neglected from the analysis. A dominant component of the biological molecules is the C-H stretch vibrational band [71]. Despite the majority, due to its weak dependence on the structural motif, it is believed to be less important for the analysis of the molecular structure and dynamics [72,73,74,75]. Moreover, the spectral features in the C-H stretch vibrational region are very congested for peptides and protein, which makes them difficult to study [39,76]. Recently, it has been proven that the C-H vibrational band involve dynamical phenomena, like chemical reaction dynamics, vibrational energy transfer, intra- and inter-molecular vibrational coupling, as well as other transient phenomena [20,77,78]. As a consequence, the C-H stretch vibrations have become important probes to reveal biological structures, especially in medical applications [79,80,81].

In reality, the C-H stretch vibrational bands in proteins are not only interacting themselves but also interact with the other vibrational bands of the protein, and reflect a reasonably strong dependency of the molecular structure. In addition, a strong anharmonicity is observed in the C-H vibrational frequency due to the intra- and inter-molecular coupling. Therefore, it is essential to consider multidimensional potential energy surfaces for a better description of the C-H vibrational bands.

**Figure 2:**
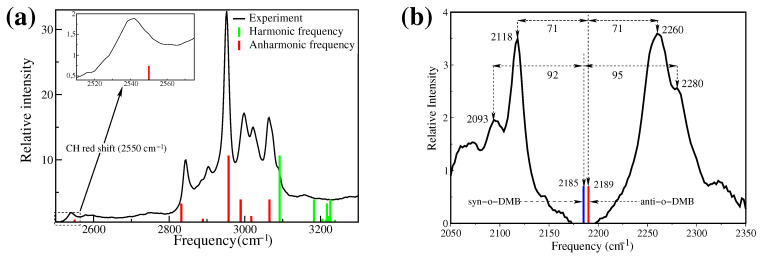
(**a**) The experimental 1D FTIR spectra (black line) of deuterated MB at the C-H absorption region. The calculated harmonic (green) and anharmonic (red) vibrational frequencies are plotted as a stick plot. The stick height represents the absorption strength of the peak. The absorption strength of harmonic frequencies is used to represent the peak strength of corresponding anharmonic frequencies. The red shift of the C-H vibration and corresponding calculated frequency are shown in the inset. (**b**) Frequency shift of C-D band due to Fermi resonance. The blue and red sticks are the calculated C-D frequencies of anti- and syn-o-DMB, respectively.

The calculation of the C-H vibrational frequencies is often troublesome because of the near degeneracy of the C-H vibrational bands and their congested vibrational pattern in infrared spectra [82,83]. For the calculation of anharmonic frequencies, it is essential to compute the energy levels and wavefunctions for the system of many coupled degrees of freedom which rise very fast with the molecular size. As a demonstration, the C-H vibrational frequencies have been calculated using 1D, as well as 2D coupling potential for deuterated methyl benzoate [84]. Very good agreement has been obtained with the experimental frequencies when C-H vibrational frequencies were calculated using 2D PESs (see Table 1). All the frequencies calculated using 1D PESs were significantly higher in value in comparison to the experimental frequencies. In particular, mode 42 is far too high for 1D PES, whereas the calculated frequency (2550 cm${}^{-1}$) using 2D PESs matches quite well with the experimental frequency (2543 cm${}^{-1}$) (see Figure 2a). However, the question is, “Why is one of the C-H frequencies surprisingly red shifted by more than 400 cm${}^{-1}$ form the usual C-H vibrational frequencies and why is harmonic and 1D anharmonic calculation unable to predict this frequency’?” To understand this unusual red shift, it is necessary to look closely at the structure of MB, possible vibrational motions of the constituent bonds, and their couplings.

**Figure 3:**
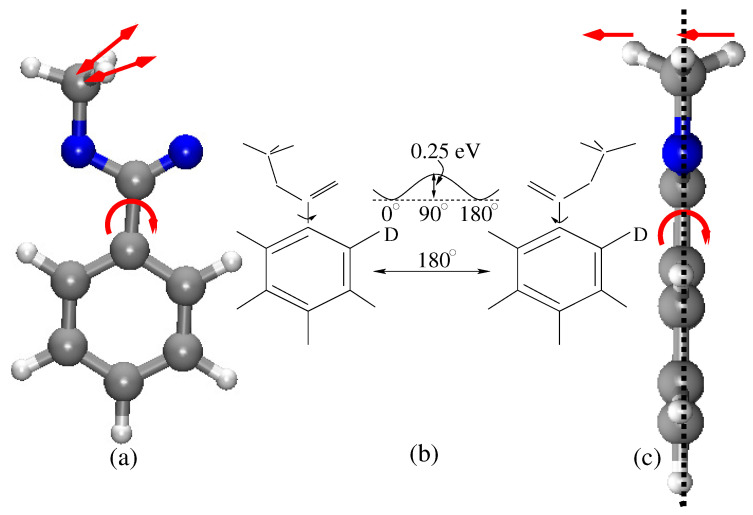
(**a**) Structure of MB, where the plane of the benzene ring is on the paper, (**b**) two stable conformers of MB and the calculated barrier height for their interconversion, and (**c**) the structure of MB, where the plane of the benzene ring is orthogonal to the plane of paper [84].

The in-plane and perpendicular views of the molecular geometries of MB are shown in Figure 3. In a close inspection of mode 42, it was found that the origin of the unexpected red shift of the C-H frequency occurred because of the vibrational coupling of the anti-symmetric C–H (two out of plane hydrogen atoms) vibration of the methyl group and the out-of-the-plane rotation of the ester group about the C–C bond w.r.t. the phenyl ring (mode 1). Due to the rotation of the ester group, one of the out-of-plane hydrogen atoms was pulled to the molecular plane, and the other out-of-plane hydrogen atom was pushed from the plane of the molecule (see Figure 3c). The force constants of the anti-symmetric C–H stretch vibrations were strongly modulated due to the bi-directional force. As a consequence, the C-H vibrational frequency of mode 42 was unexpectedly red-shifted. Since mode 1 and mode 42 are strongly coupled, the harmonic and the 1D anharmonic analysis, which do not account for the coupling in the calculation, failed to describe the red shift of the C-H frequency correctly. On the other hand, in the pairwise PES calculations, the coupling effects were considered. As a result, the anharmonic frequency of mode 42, calculated using 2D PES, agreed well with the experimental result [84]. Therefore, it is absolutely necessary to consider the coupling potential (2D PES) for a better description of the C-H vibrational band in protein vibrational spectroscopy.

### 3.3. Isotope Labeling

It is well-known that the vibrational spectra of protein are congested and it is very difficult to assign the vibrational peaks observed in the spectra. Isotope labeling in a specific site of the protein makes the assignment easier [85]. In particular, the substitution of hydrogen by deuterium is very efficient, since the change of effective mass is double, and as a result, the absorption peak is shifted significantly. For example, hydrogen substitution by deuterium is performed for MB, and anharmonic vibrational spectra have been calculated using multidimensional PES and the VSCF method [62]. Two isotopomers are formed when one of the hydrogen atoms in the ortho position (β-hydrogen) of the benzene ring is substituted by deuterium. The schematic of the two isotopomers, syn-ortho-deuterated methyl benzoate (syn-o-DMB) and anti-ortho-deuterated methyl benzoate (anti-o-DMB) are depicted in Figure 3b). The C-D stretch vibrational frequency has been calculated using 1D PES, as well as considering multidimensional potentials in VSCF expansion. The calculated C-D frequency using 1D PES is far from the experimental value. On the other hand, the calculated frequency using 2D PES is well-supported by the experimental results (mode 44 in Table 1). The calculated frequencies for the isotopomers syn-o-DMB (2185 cm${}^{-1}$) and anti-o-DMB (2189 cm${}^{-1}$) are located at the middle of two distinguishable experimental peaks (see Figure 2b). In general, the C-D vibrational frequency is isolated from the remaining spectral feature of the molecule; moreover, it exhibits a reasonably strong coupling with a few other vibrational modes. The pair coupling PES accounts for those couplings in the calculation, and as a result, the calculated frequency is very close to the experimental frequency. In the experimental spectra, two distinguishable peaks are observed at 2118 cm${}^{-1}$ and 2260 cm${}^{-1}$. These two peaks are symmetrically (71 cm${}^{-1}$) segregated from the calculated anharmonic C-D stretch vibrational frequency of anti-o-DMB at 2189 cm${}^{-1}$. A careful investigation reveals that the overtone of the in-plane phenyl ring deformation (1097 cm${}^{-1}$) is closer to the fundamental C-D anharmonic frequency of the anti-o-DMB. As a consequence, there is a possibility of the Fermi resonance occurring. Due to the Fermi resonance, the fundamental C-D vibrational peak splits into two peaks which are uniformly shifted from the original peak position. In general, at room temperature, both syn- and anti-o-DMB are present in solutions with slightly different C-D frequencies. Therefore, there is a possibility for more Fermi resonances to occur. Two more prominent absorption peaks are observed at 2093 cm${}^{-1}$ and 2280 cm${}^{-1}$ in the experimental spectra. They are assigned as the Fermi resonance frequencies of the overtone of the in-plane phenyl ring deformation and C-D vibration (2185 cm${}^{-1}$) of syn-o-DMB isotopomer.

It is clear from the above examples that the frequencies calculated using 1D PES differ largely from the experimental frequencies. Molecular vibrations are not decoupled from each other. As 1D PES does not consider the vibrational couplings, the description of the PES is far from reality. Therefore, it is essential to consider the multidimensional coupling PESs in the frequency calculation. The inclusion of 2D PESs already improves the frequency calculation significantly and calculated frequencies are very close to the experimental frequency. The inclusion of higher-order coupling PES was expected to improve the frequency calculation; however, in many cases, it is not cost-effective. As a compromise, a reasonably good agreement was achieved when an anharmonic vibrational frequency was calculated using 2D PES, provided strong coupling potentials were accounted for in the calculation.

Up to now, the necessity of 2D PESs in the anharmonic frequency calculation has been discussed, and it has been established that the inclusion of strongly coupled vibrational modes is essential for the accurate calculation of the anharmonic vibrational frequencies. In the following sections, it will be shown how 2DIR spectroscopy readily provides the coupling behavior of different vibrational bands and the structural dependencies of the different bands. In addition, the time evolution of molecular structures will be explained with the time sequence of 2DIR spectra.

## 4. Experimental 2D Vibrational Spectroscopy

**Figure 4:**
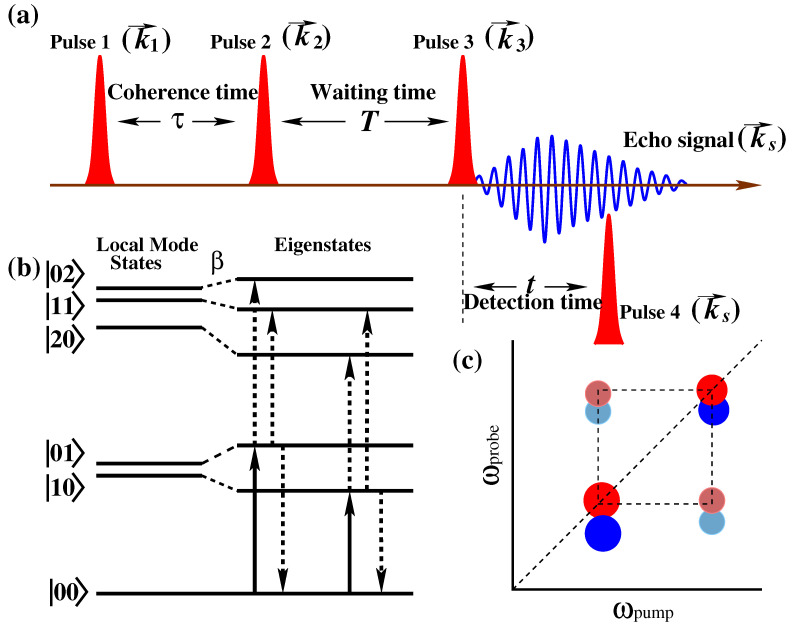
(**a**) Pulse sequence of the photon echo experiment. (**b**) Schematic diagram of two coupled oscillators. Before coupling, vibrational states are depicted as “local modes” and after coupling as “eigenstates”. Vibrational transitions involved in 2DIR spectroscopy of the pair oscillators are depicted by arrows where solid arrows represent the pump processes and probes are represented by dotted arrows. (**c**) The schematic of 2DIR spectral features. The red and blue peaks represent signals with opposite signs.

In the last two decades, there has been exceptional development of 2DIR methodology theoretically as well as experimentally from its first report in 1998 [35]. It has gone through several technological milestones of developments, for example, hole-burning [35,86,87], heterodyned four-wave mixing in box-cars geometry [88,89,90,91,92], three-pulse photon echoes in pump-probe configuration [93,94], acousto-optic pulse shaping [95,96], and so forth. All these methods contribute to improving the versatility of the experimental technique. Irrespective of the techniques, all the above-mentioned techniques share a few common features. A sequence of excitation pulses excite a molecular vibrational band of interest and probe the vibrational frequency change to another vibrational band. This is analogous to considering pair potential (2D PES) in an anharmonic vibrational frequency calculation in the theoretical approach. The molecular responses were plotted as a two-dimensional map with two frequency axes. Molecular signatures were encoded in peaks with different shapes, intensities, and positions in the two-dimensional map, as shown in Figure 4c.

### 4.1. Two-dimensional IR Spectroscopic Technique

2DIR spectroscopy is third-order nonlinear spectroscopy where three ultrafast infrared pulses having controlled time intervals between them are successively interact with a set of vibrational transitions in a multilevel vibrational system [97]. If the interactions take place at the absolute times *t*${}_{1}$, *t*${}_{2}$, and *t*${}_{3}$, then the emitted electric field by the system, *E*${}^{\left(3\right)}$(*t*) is written as

(2) $$\begin{array}{ccc} E^{\left(3\right)}\left(t\right) & = & {\left(\frac{i}{h}\right)}^{3}\int dt_{3}\int dt_{2}\int dt_{1}\sum R_{n}\left(t_{1},t_{2},t_{3}\right)E_{3}\left(t-t_{3}\right)e^{-i\omega (t-t_{3})}\\ & \times & E_{2}\left(t-t_{3}-t_{2}\right)e^{-i\omega (t-t_{3}-t_{2})}E_{1}^{*}\left(t-t_{3}-t_{2}-t_{1}\right)e^{i\omega (t-t_{3}-t_{2}-t_{1})}\\ & \times & e^{i(+k_{3}+k_{2}-k_{1})}e^{i(+\phi_{3}+\phi_{2}-\phi_{1})} \end{array}$$

where $R_{n}\left(t_{1},t_{2},t_{3}\right)$ is the third-order system response (which holds the necessary structural information of the system of interactions), $E_{n}$ are the envelopes, $k_{n}$ are the wavevectors, and $\phi_{n}$ are the phases of the $n^{th}$ laser pulse. The summation in Equation (2) is the overall possible rephasing and non-rephasing signals which arise from Feynman pathways [23,36,98]. The electric fields are generated into phase-matched directions $k_{s}=+k_{3}+k_{2}-k_{1}$ (echo signal) and $k_{s}=+k_{3}-k_{2}+k_{1}$ (nonrephasing signal). The emitted echo signal requires a phase-sensitive detection [99]. This can be achieved by heterodyning $E^{\left(3\right)}\left(t\right)$ with a phase-locked fourth ultrafast laser pulse with the identical wavevector as the emitted field. This fourth laser pulse is commonly called the local oscillator [100]. The detected signal is denoted as

(3) $$\begin{array}{c} S\left(\tau ,T,t\right)=\int dt_{4}{\left|E^{\left(3\right)}\left(\tau ,T,t_{4}\right)+E_{4}\left(t-t_{4}\right)\right|}^{2} \end{array}$$

where $\tau =t_{1}$, $T=t_{1}+t_{2}$ and $t=t_{1}+t_{2}+t_{3}$ are the relative time between the pulses. Here, it is noted that absolute times are frequently used in the derivation of $E^{\left(3\right)}\left(t\right)$; however, relative times are more inherent in the discussion of pulse sequences (see Figure 4a).

A schematic of the pulse sequences, where interaction with two coupled oscillatory systems and the 2DIR spectra are shown in Figure 4. In order to understand the 2DIR spectra, the comprehensive character of the molecular populations and coherences due to the interaction of the ultrashort infrared pulses and the echo signal emitted after the third infrared pulse must be known. Let us consider, initially, the molecule is in the ground state (|00>). After interaction with the first laser pulse, the system propagates in a vibrational coherence. Practically, the first pulse labels the initial structure of the molecule by setting its initial frequencies. The second pulse turns on the reaction time period T${}_{w}$, during which the molecule undergoes changes to different population dynamics, such as vibrational relaxation to the ground state, orientational relaxation of the molecular system, and so forth. The time between the first and second infrared pulses is called the coherence time. In practice, experimentally, it is scanned over a time period of several hundreds of fs with time-steps controlled to an accuracy of a small fraction of a cycle, typically 1 to 3 fs. After waiting for a while, the third pulse is applied. The interval between the second and the third pulses is called population time T${}_{w}$. It is also known as the waiting time. An echo signal is generated after the third pulse excites the molecule. The vibrational echo signal contains the information of the molecule at the moment the third pulse interacts with the system. Finally, the echo signal is detected with the help of a local oscillator (LO) [100].

**Figure 5:**
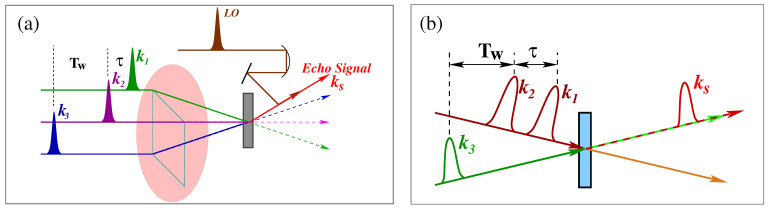
Schematic layouts of 2DIR experiments: (**a**) four-wave mixing method in box-cars geometry and (**b**) partly collinear method as like pump-probe geometry.

The experimental techniques of different 2DIR methods have been documented in several publications [35,36,96]. Here, only two extensively used experimental techniques are described briefly with their advantages and disadvantages. In the early stage of its development, four waves mixing in box-cars geometry seems a very promising method to explore the three-dimensional molecular structure and dynamics. In this experiment, three ultrashort pulse infrared beams are focused into the sample non-collinearly, as if they are focusing from three corners of a square pattern (see Figure 5a). The spatial and temporal overlaps of the three non-collinear beams are extremely difficult, especially when invisible IR light is used. In this experimental arrangement, the echo signal is generated and directed ($\vec{k_{s}}$) in a direction as if it is coming from the fourth corner of the square pattern. A fourth IR pulse (LO) is used for the heterodyne detection of the echo signal [101]. Again, the overlap of LO with the echo signal is cumbersome. On the other hand, photon echo in pump-probe geometry is easier to establish, since practically only two laser beams are required to focus into the sample. Of course, it is also a four-wave mixing process. In this experimental arrangement, two of the excitation pulses travel collinearly. These two pulses are generated either by using acousto-optic pulse shaping [96,102] or by overlapping two individual IR beams [94,103]. These two collinear beams meet the third excitation beam in space and time into the sample with a small angle, as like a pump-probe experimental arrangement (see Figure 5b). Practically in this experimental arrangement, only two beams are needed to focus into the sample in space and time, which is much easier than focusing three beams in box-cars geometry. The foremost advantage of the pump-probe geometry is that the echo signal is generated in the same direction as the third laser pulse. As a result, the third pulse itself acts as a LO, and the self-heterodyne detection is performed. Moreover, the pulse pair (first and second pulses) are collinear and indistinguishable with respect to the time ordering, therefore, both rephasing and non-rephasing coherence pathways contribute to the signal. As a result, data acquisition is faster compared to the experiment performed by the box-cars geometry experimental scheme. Despite many advantages, there is a technical disadvantage of the experimental arrangement in pump-probe geometry. As the third excitation beam acts as LO, the intensity of LO cannot be controlled independently for the optimization of the heterodyne of the signal. However, many experimental results demonstrated that there is an adequate signal-to-noise ratio for rapid acquisition of 2DIR spectra [78,103,104].

### 4.2. Two-dimensional IR Spectra

In 2DIR spectroscopy, two fundamental pieces of structural information are available, for example, vibrational couplings and spectral lineshapes. Vibrational couplings are evaluated by the strength and positions of diagonal and cross-peaks in 2DIR spectra. On the other hand, lineshapes are evaluated by the shapes of the diagonal peaks. The schematic of 2DIR spectra is shown in Figure 4c. The diagonal peaks carry out similar information like one-dimensional IR (1DIR) spectra; however, the shape of the peaks reflects the broadening characteristics of the spectra. Cross-peaks in 2DIR spectroscopy carry the most valuable information about the molecular structure. The coupling between different vibrational modes is observed directly from the cross-peak position, shape, and strength, whereas in linear spectroscopy, coupling can only be guessed by modeling the linear spectra. For example, the amide modes of a peptide and protein display several vibrational transitions. These vibrational transitions are often modeled with a set of coupled modes or vibrational excitons [105]. Therefore, in 1DIR spectroscopy, structural information can only be speculated by using such a model. On the contrary, the presence of the cross-peaks in 2DIR spectra come up with a model-free confirmation if different vibrational modes are sensing each other and provides a quantitative measure of the coupling by the cross-peak position, shape, and strength.

### 4.3. Two-dimensional IR Spectra of Amide **I** Band

**Figure 6:**
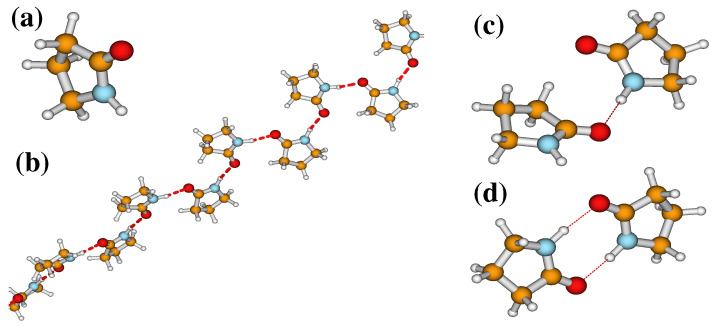
Molecular structure of 2-Pyrrolidinone and its possible hydrogen bonded oligomers. The hydrogen bond is represented by a red dashed line between molecules. (**a**) Monomer, (**b**) single hydrogen-bonded oligomer (SHBO), (**c**) single hydrogen-bonded dimer (SHBD), and (**d**) doubly hydrogen-bonded dimer (DHBD).

In protein, amide **I** bands are not only coupled with the adjacent vibrational bands, but also coupled with vibrational bands located in different amino acid residues [105,106,107,108]. In addition to that, it may also be coupled with the environment [109,110,111,112]. As a result, amide **I** spectra in protein are highly complex [105,113,114,115]. To reveal the structural sensitivity of the amide **I** band, 2DIR spectra of a relatively less complicated small model molecule have been presented here, where only a few couplings are involved. The amide **I** band in 2-Pyrrolidinone is well-known for the formation of hydrogen bonding with the amide **A** band of another 2-Pyrrolidinone molecule. This can be used as a perfect model to understand the structural sensitivity of the amide **I** band. The molecular structure of 2-Pyrrolidinone is depicted in Figure 6. Interestingly, 2-Pyrrolidinone not only makes a single hydrogen-bonded dimer (SHBD), but also makes doubly hydrogen-bonded dimer (DHBD) and single hydrogen-bonded oligomers (SHBO) of different sizes of molecular chains (see Figure 6). This verity of hydrogen bonding provides an enormous opportunity to study different hydrogen bonding characteristics of the protein.

**Figure 7:**
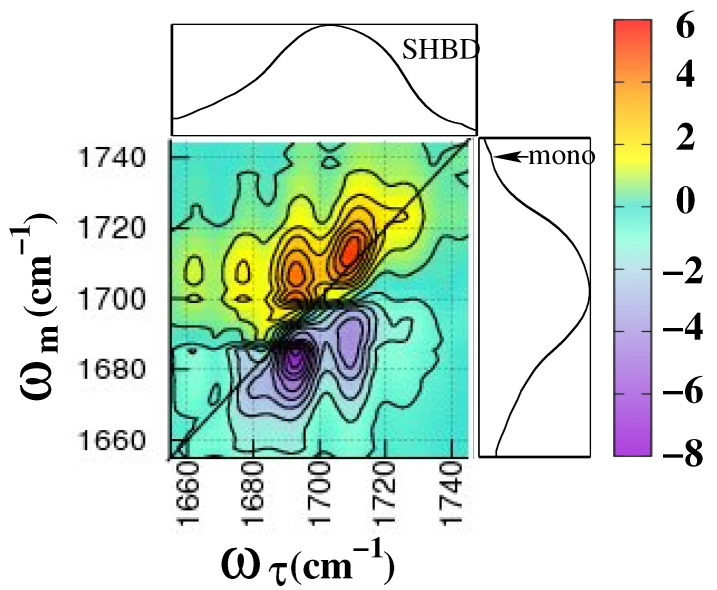
2DIR spectra of 2-Pyrrolidinone in CCl${}_{4}$ at a population time of T${}_{\omega }$ = 200 fs. Two-dimensional spectra are embedded with 1DIR spectra from the top and right side. The red contours in 2DIR spectra are positive-trending and the blue are negative-trending. The excitation frequency is plotted in the horizontal axis and the detection frequency is plotted in the vertical axis.

The amide **I** vibrational band has been extensively studied for several decades for the structural analysis of protein using vibrational spectroscopy [28,116,117,118,119]. It is no wonder that it is also studied by 2DIR spectroscopy to a great extent [7,24,98]. In short, it is explained here. The 2DIR spectra of the amide **I** band of 2-Pyrrolidinone are depicted in Figure 7. For a better understanding of the peak positions in the 2DIR spectra, 1DIR spectra are embedded on the top and the right side. Two prominent red peaks are observed on the diagonal. The corresponding 1D spectra seem featureless but unexpectedly broad. However, with careful investigation, it is found that a small hump is visible at the foot of the blue side of the spectra (at 1740 cm${}^{-1}$), which is assigned as an amide **I** band from a 2-Pyrrolidinone monomer [78]. In normal thermal conditions, a few monomers exist in the solution; as a result, the absorption strength of amide **I** from monomers is very low and hardly visible in 1DIR, as well as 2DIR spectra. However, at around (1722, 1740) cm${}^{-1}$ and (1740, 1722) cm${}^{-1}$, two noticeable cross-peaks are observed. These cross-peaks are evidence of the hydrogen bond-breaking process of SHBD to form two monomers and vice versa. The second hump (∼1710 cm${}^{-1}$), which is hard to distinguish in 1DIR spectra, prominently appears on the diagonal in 2DIR spectra. This peak is assigned as the amide **I** band of SHBO. The amide **I** peak from DHBD is supposed to appear at around 1690 cm${}^{-1}$; however, due to the strong absorption by water vapor in the experimental setup [94], this pick seems quite weak and also covered by black lines (see Figure 7). Nevertheless, a strong cross-peak at around (1690, 1710) cm${}^{-1}$ indicates the hydrogen bond breaking and making the mechanism among DHBD and the polymeric chain (SHBO). The negative two peaks (below the diagonal) are due to the excited state absorption of amide **I** bands of the polymeric chain and DHBD.

### 4.4. Observation of Hydrogen Bond Breaking and Making

Biological activities are predominantly controlled by hydrogen-bonding of amide bands among different peptide units, as well as with surrounding molecules [120,121]. In particular, the hydrogen bond stabilizes the protein structure, and therefore controls biological processes like molecular recognition, self-assembly, substrate binding, protein folding, and so forth [122,123,124]. Particular hydrogen-bonding strength and configuration have a notable impact on the protein structure and dynamics [125,126,127,128]. For example, the hydrogen-bonding stretch and twist modes strongly influence the vibrational dynamics of the molecules [129,130]. Therefore, to understand the protein structure and dynamics, it is an essential task to understand the hydrogen-bonding characteristics in detail. The amide **A** band of 2-Pyrrolidinone has a strong affinity to form hydrogen bonding, and its vibrational frequency is strongly modified. Therefore, it can be used as an appropriate model to understand the hydrogen-bonding characteristics in detail.

**Figure 8:**
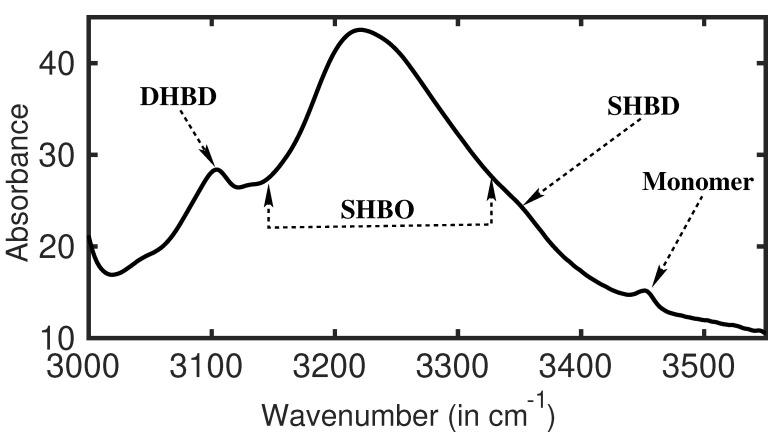
1D absorption spectra (FTIR) of 2-Pyrrolidinone in carbon tetrachloride is collected at the amide **A** spectral region.

The experimental 1DIR spectra in the amide **A** stretch vibrational spectral region of 2-Pyrrolidinone in carbon tetrachloride (20% by volume concentration) is depicted in Figure 8. It is essentially the N-H stretch vibration of 2-Pyrrolidinone. In general, it is expected to be present at the spectral position of 3450 cm${}^{-1}$ [33]. A considerably low intense narrow peak is noticed at 3450 cm${}^{-1}$, although a much stronger one might be expected. Rather, a strong and very broad (FWHM ca. 165 cm${}^{-1}$) absorption peak is noticed at around 3212 cm${}^{-1}$. This peak appears with a few shoulder peaks. A concentration-dependent FTIR measurement and theoretical calculations confirm this broad peak as the amide **A** stretch vibrational absorption from the single hydrogen-bonded oligomers of 2-Pyrolidinone [39]. In general, at normal thermal condition, a large distribution of molecular chains of SHBO are possible in the solution. The vibrational frequency of the amide **A** band of SHBO is strongly modulated by the number of molecules present in the oligomers. As a consequence, the amide **A** band from different-sized SHBO possess different absorption frequencies, and a broad absorption peak is observed. The smoothness of the spectral feature indicates that the distribution of SHBO chain size is regular and there is an optimal chain size which is most probable and shows a strong absorption at around 3212 cm${}^{-1}$. It is also necessary to mention here that with increasing oligomer size, amide **A** frequency is gradually red-shifted. It is clear from the plot that the broad amide **A** peak is highly asymmetric in nature. The slope of the spectral envelopes practically specifies the distribution pattern of the oligomers. The peak is slowly grown from the right side, which reflects that the population of oligomers with different chain size slowly increases to an optimal length. On the contrary, the population of longer chains drops very fast (left side of the peak) from the optimal chain length. As mentioned before, this broad peak appears with few shoulder peaks, one of them being identified at around 3360 cm${}^{-1}$ (see Figure 8). This small hump is identified as the amide **A** absorption of SHBD. A slightly elevated population of SHBD is expected. The reason for this elevated population of SHBD is explained in the following section. A relatively prominent peak at 3106 cm${}^{-1}$ is identified as the amide A absorption from DHBD. Since two hydrogen bonds are formed among two 2-Pyrrolidinone molecules in DHBD, the bonds are generally quite stable and stronger than SHBO. The strong hydrogen bonds also strongly modulate the N-H vibration, and as a result, the amide **A** from DHBD is strongly red-shifted by almost 350 cm${}^{-1}$ from the monomer’s amide **A** band spectral position. All these explanations are some short of speculations. The assignments are clearer in 2DIR spectra, as explained below.

**Figure 9:**
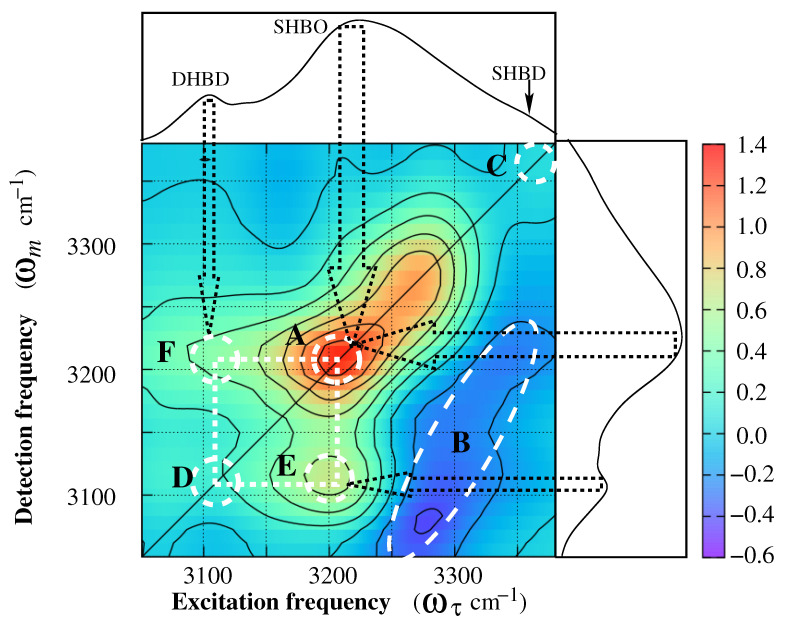
2DIR photons echo real spectra of 2-Pyrrolidinone at a population time of T${}_{w}$ = 600 fs. The spectra are collected at the N–H stretch vibrational region and plotted along with 1DIR spectra (FTIR) embedded on the right and top side of 2DIR spectra. The red colour contours are positive-trending and the blue colour contours are negative-trending. The x-axis is the Fourier-transformed τ-axis and the y-axis is the monochromator axis ($\omega_{m}$ axis). Both the linear and two-dimensional spectra are acquired at room temperature.

2DIR spectra of 2-Pyrrolidinone at a population time of T${}_{w}$ = 600 fs are presented in Figure 9. The spectra are collected at the amide **A** stretch vibrational region. The pump frequency ($\omega_{\tau }$) is plotted on the horizontal axis and the probe frequency ($\omega_{m}$) is on the vertical axis. To make the understanding easier, 1DIR spectra are embedded on the top and the right side of the 2DIR spectra. The dotted arrows from the 1DIR peak position to the 2DIR peak, practically map the peak positions of two representations. A strong positive peak (“A”) is found on the middle of the 2DIR spectra whose epicenter is at around 3225 cm${}^{-1}$ (see Figure 9). The peak is exceptionally broad, asymmetric, and elongated along the diagonal. The intensity and the shape of the peak are not uniform. The intensity distribution of this positive peak precisely follows the intensity pattern of the 1DIR spectral peak, which is assigned as the hydrogen-bonded amide **A** stretch vibrations of SHBO. This positive peak appeared because of the ground-state bleach and excited-state emission of the hydrogen-bonded amide **A** stretch vibrations of SHBO. The corresponding excited state absorption peak appeared as a negative peak (blue peak, “B”), parallel with the diagonal amide **A** peak. The peak is marked with a dotted oval. The lower side of the negative peak is slightly deformed due to the presence of an off-diagonal positive peak at (3200, 3106 cm${}^{-1}$). The origin of this off-diagonal positive peak is explained in the following section.

Two more positive peaks are expected on the diagonal, one from SHBD and another from DHBD. The diagonal peak for SHBD is expected at around ($\omega_{\tau }=\omega_{m}=3360$ cm${}^{-1}$). Indeed, a significantly low intense peak (“C”) is present on the diagonal (at the top right corner of the 2DIR spectra). This peak originated because of the ground-state bleach and excited-state emission of the amide **A** band of SHBD. The other diagonal peak which was supposed to originate from the amide **A** peak for DHBD was expected to appear at around ($\omega_{\tau }=\omega_{m}=3106$ cm${}^{-1}$). The position of this peak was marked with a white dotted circle (“D”). Due to the presence of surrounding high intense peaks, it was hardly recognisable. However, two positive off-diagonal peaks at around (3210, 3106 cm${}^{-1}$; “E”) and (3106, 3210 cm${}^{-1}$; “F”) confirm the existence of this diagonal peak. Along with the diagonal peaks “A” and “D”, the off-diagonal peaks (“E”, “F”) form a square pattern (white dotted square in Figure 9). This square pattern demonstrates the hydrogen bond making and breaking dynamics of DHBD and SHBO and confirm the presence of the diagonal peak “D”. In one process, two molecules from the end of an oligomer breaks the hydrogen bond and separate from the long chain. Finally, these two molecules (practically SHBD) form the second hydrogen bond among themselves, and a DHBD is formed. This hydrogen bond breaking and making process leads to the positive off-diagonal peak “E”. In the second process, out of two hydrogen bonds in DHBD, one of the bonds breaks. This breaking process leads to a SHBD. This SHBD finally comes closer to an oligomer and makes another hydrogen bond with the oligomer to form a longer chain. This breaking and making processes leads to the positive off-diagonal peak “F”. According to the square pattern, it is understandable that the oligomers with the optimal chain lengths are more favorable to the hydrogen bond making and breaking processes. The making and breaking processes of hydrogen bonding are bi-directional. In general, these bi-directional processes are stabilized with the temperature. It is noted here that each process of these bi-directional processes produces some intermediate SHBD. As a consequence, on average, there are more SHBD than expected, which leads to a recognizable positive peak on the diagonal at 3360 cm${}^{-1}$, as well as at 1DIR spectra. The hydrogen bond breaking and making processes, and the presence of more excess SHBD than expected are not possible to understand from 1DIR spectra. Thus, 2DIR spectra yield a better understanding of the molecular geometry as well as the dynamical processes occurring in the system. There are further advantages of the 2DIR spectroscopy; it is allowed to monitor the dynamical processes occurring in the molecule. The following example will show how 2DIR spectroscopy allows monitoring of the dynamical processes.

## 5. Dual Frequency 2DIR Spectroscopy

Up to now, 2DIR spectra have been presented for a single vibrational band of a molecule. However, the major advantage of 2DIR spectroscopy is the simultaneous observation of two or more different molecular vibrations and their couplings. The availability of a tunable (2.5 to 20 μm wavelength) laser system [131] allows to utilise the versatility of the 2DIR spectroscopy to monitor several vibrational bands and their interactions simultaneously. Such an experiment can be performed by constructing the pulse sequences using infrared lights having different central wavelengths. When two different wavelengths of infrared lights are used in the experiment, the experimental technique is called dual-frequency 2DIR spectroscopy [96,132]. The dual-frequency experiments not only provide intermode couplings, but also provide additional structural constraints [78,94,106,133].

The intermode coupling between amide **I** and amide **A** is a common phenomenon in protein dynamics [1]. It has been already explored extensively by 2DIR spectroscopy by many researchers [24,134]. The C-H band, which is a major component of the proteins and peptides, is neglected in protein research, although it carries out important structural information. In reality, due to the congested spectral signature and presence of degenerate modes, experimental as well as theoretical investigations of the C-H vibrational modes and their couplings are extremely difficult [84]. In addition, the weak absorption of the C-H vibrational bands in comparison with the strong absorption of the amide bands makes such experiments challenging to study with regard to the coupling between the amide and C-H vibrations. Recently, it has been reported that amide **I** and C-H bands are coupled with a noticeable coupling constant [78,135]. Amide **I** and C-H vibrational coupling dynamics are presented in the following sections.

**Figure 10:**
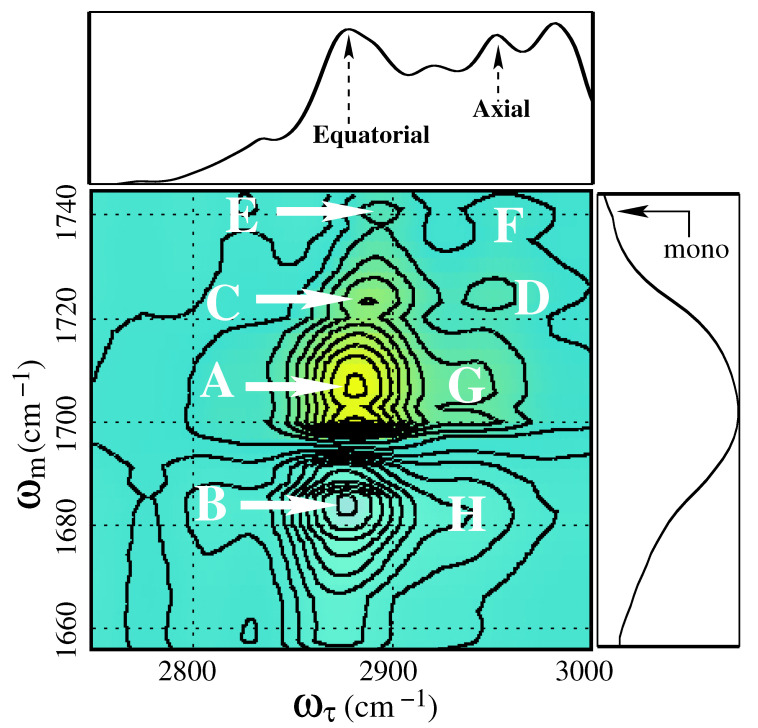
Two-dimensional vibrational photons echo real spectra at the CHCO coupling region of 2-Pyrrolidinone at a population tine $T_{w}=3000$ fs. Spectra is taken by pumping at the C-H vibrational modes and probing at the C=O vibrational mode. The 1DIR spectra at the C-H vibrations region is embedded on top, and the C=O spectral region on the right side of 2DIR spectra for a better understanding of the peak position. Two-dimensional data have been normalized to the highest peak intensity.

To study the C-H and amide **I** vibrational coupling, it is necessary to perform the dual-frequency experiment. In the experiment, one of the excitation pulses is set to the C-H vibrational region, and another excitation pulse is set to the amide **I** vibrational region. The 2DIR spectra in the C-H and amide **I** cross-vibrational region are depicted in Figure 10. To map the cross-peak positions, the 1DIR spectra in the C-H vibrational region are embedded on the top of the 2DIR spectra, and linear absorption spectra in the amide **I** region is embedded on the right. Six C-H vibrational absorption peaks were found in the linear absorption spectra, as expected. Among six C-H absorption peaks, the “axial” and the “equatorial” C-H vibrational modes are labeled in the 1DIR spectra. These two C-H vibrations take part in the crucial dynamical processes of the molecule. As mentioned before, the amide **I** spectral region apparently looks featureless; however, with a careful investigation, the monomer and the SHBD peaks are identified in the 1DIR spectra.

**Figure 11:**
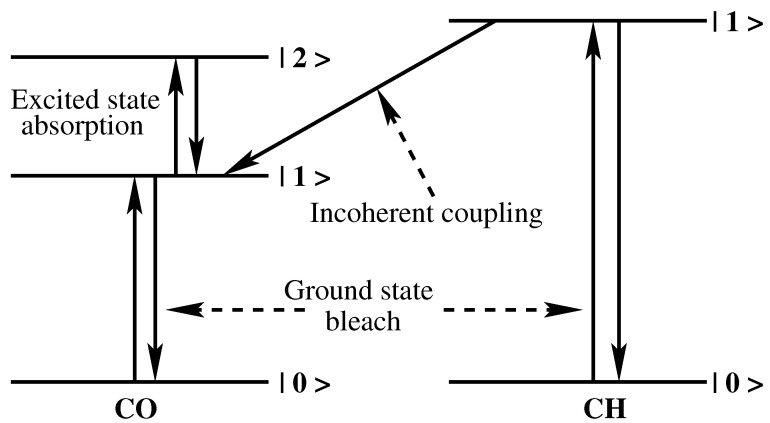
Incoherent coupling: Relaxation of energy from the first excited energy level of the C-H stretch vibrational band to the first excited energy level of the amide I band.

The characteristic of the cross-peak in 2DIR spectroscopy is that the intensity of the peak grows with the increase of waiting time (T${}_{w}$). In a short waiting time, off-diagonal peaks are hardly visible in the 2DIR spectra. To discuss the coupling behavior of the C-H and the amide **I** vibrations, the 2DIR spectra at 3 ps have been chosen (Figure 10). It is a relatively long waiting time with respect to the vibrational lifetime of the C-H and the amide **I** bands. By this time, most of the off-diagonal peaks reach their saturation. A prominent positive peak (“A”) is observed at the middle of the spectra (2880, 1710 cm${}^{-1}$) in Figure 10. The position of the peak indicates that it originated due to the coherent coupling between the equatorial C-H vibration and the amide **I** vibration from the SHBO. A negative peak with a similar shape is observed just below the above-mentioned positive peak (2880, 1685 cm${}^{-1}$). The peak is marked as “B” in Figure 10. This negative peak originated due to the incoherent coupling of the equatorial C-H vibrational band and the amide **I** band. In this process, the vibrational energy of the C-H mode is transferred to the amide **I** mode. The energy transfer mechanism is depicted in Figure 11. The first excited state (|1>) of the C-H vibration is higher in energy than the first excited state of the amide **I** vibration. Therefore, there is a chance of energy transfer from the excited-state C-H band to the excited-state amide **I** band. After absorbing the energy, the amide **I** band goes to the second (|2>) excited state. This excited-state absorption appears as a negative off-diagonal peak in 2DIR spectra.

The second prominent positive off-diagonal peak (“C”) is observed at the spectral position (2890, 1725 cm${}^{-1}$). This peak originated due to the coherent coupling between the C-H band at 2890 cm${}^{-1}$ and the amide **I** band of SHBD. Despite the similar peak strength of the C-H bands at 2880 cm${}^{-1}$ and 2890 cm${}^{-1}$, the strength of the cross-peaks at “A” and “C” differs significantly. The lower strength of the peak “C” is due to the significantly lower population of the SHBD than the SHBO in the sample. The C-H vibration with absorption frequency 2950 cm${}^{-1}$ shows a considerable coupling with the amide **I** band of the SHBD. This off-diagonal peak appears at the spectral position (2950, 1725 cm${}^{-1}$). The peak is marked as “D”. Another C-H vibration appears at the spectral position of 2920 cm${}^{-1}$, and shows a reasonable coupling with the amide **I** band of the oligomers. The coupling peak is observed at the spectral position (2920, 1710 cm${}^{-1}$) and denoted as “G”. The corresponding negative peak (“H”) is observed at (2920, 1685 cm${}^{-1}$). This peak appears due to the incoherent coupling among the amide **I** and axial C-H vibrational bands of the SHBO. Two more recognizable off-diagonal peaks appear at the top of the 2DIR spectra, marked as “E” at (2890, 1740 cm${}^{-1}$) and “F” at (2950, 1740 cm${}^{-1}$). In linear absorption spectra in the amide **I** spectral region, a very low, intense absorption peak is observed at around 1740 cm${}^{-1}$. This peak is labeled as “mono” since this absorption peak originated due to the absorption of the amide **I** band of 2-Pyrrolidinone monomer. The peaks “E” and “F” originated because of the C-H and C=O vibrational coupling of the 2-Pyrrolidinone monomer. Apparently, the amide **I** absorption peak of 2-Pyrollidinone monomer in 1DIR absorption spectra are imperceptible; however, the appearance of the off-diagonal peaks “E” and “F” in 2DIR spectra are significantly prominent. The presence of these two off-diagonal peaks confirm the presence of monomers in the sample.

Practically all the C-H vibrations in 2-pyrrolidinone are modified due to the coupling with the C=O stretch vibration. However, the modulation of vibrational frequencies of the C-H bonds is not possible to realise from 1DIR spectra. The off-diagonal peaks in 2DIR spectroscopy are the direct evidence of the coupling. By extracting the individual coupling constant of each C-H bond with amide **I**, a much accurate structure of the molecule can be estimated. Using the same experimental procedure for proteins, more accurate structural information can be extracted.

## 6. Watching Molecular Motion

**Figure 12:**
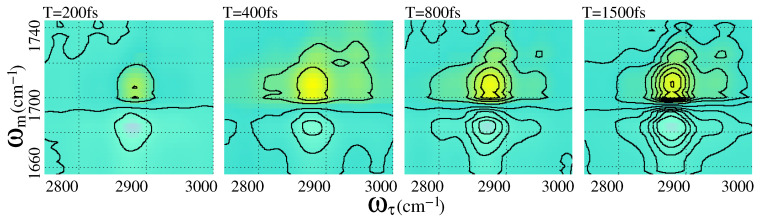
2DIR spectral snapshot of 2-Pyrrolidinone at a waiting time of 200 fs, 400 fs, 800 fs, and 1500 fs. All peaks are normalized with respect to the highest intense peak in Figure 10. At an early waiting time, off-diagonal peaks are not prominent. With the development of the waiting time, the off-diagonal peak intensity grows and reaches saturation.

**Figure 13:**
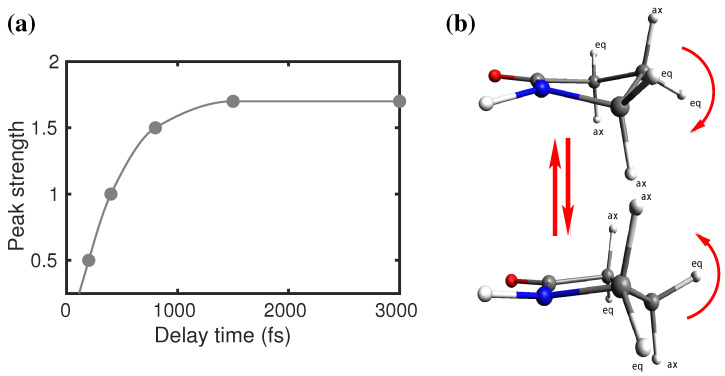
Waiting time (T${}_{w}$) vs. peak intensity for the cross-peak at position (2880, 1710 cm${}^{-1}$). The peak intensity grows very fast at an early waiting time and practically reaches saturation at a waiting time of 1.5 ps.

The major advantage of 2DIR spectroscopy is the ability to extract dynamical information of the molecule in real-time by taking the time trace of the 2DIR spectra. This unique feature of the 2DIR spectroscopy allows to observe the molecular motion in real-time. For a demonstration, the time-dependent coupling patterns are explained here by considering the 2DIR snapshots of the CHCO cross-vibrational spectral region at different waiting times. Five snapshots are presented here considering a systematic time sequence, for example, 200 fs, 400 fs, 800 fs, 1500 fs, and 3000 fs. The first four snapshots (2DIR spectra) are depicted in Figure 12. The fifth snapshot (at a waiting time of 3 ps) is already explained in the previous section. All the off-diagonal peaks in different snapshots are normalized with the highest peak intensity of Figure 10. At an early waiting time (200 fs), the CHCO spectral region is practically underpopulated, and only “A” and “B” peaks appeared in the spectra with very low intensity. With the increase of waiting time, the peak intensity of “A” grows very fast at the beginning, and practically reaches saturation at 1.5 ps. The variation of the peak intensity of “A” is plotted as a function of the waiting time in Figure 13a. An exponential increase of peak intensity is observed, which is the property of the off-diagonal peak in 2DIR spectroscopy. The calculated time constant of this off-diagonal peak is about 1 ps. A similar exponential increase of off-diagonal peak intensity is reported for metal carbonyl [136,137]. The negative off-diagonal peak “B” gradually decayed with the increase of waiting time as contrary to the positive peak “A”. This negative peak is saturated to its lowest intensity at around 1.5 ps.

With the increase in waiting time, the number and strength of the off-diagonal peaks increase furthermore in the 2DIR spectral snapshots. For example, at the waiting time of 400 fs, the off-diagonal peak (“D”) appears in the spectra. In the same snapshot, the peak “C” also appears; however, the intensity of the peak is considerably low. The coherent (“G”) and incoherent (“H”) coupling peaks also appear in this snapshot. With further waiting, at 800 fs, all the peaks which previously appeared gained their spectral strength and became prominent in the spectra. The cross-peak (“E”) from the monomer appears in this snapshot; however, the other coupling peak (“F”) from the monomer does not yer appear in the spectra. With further waiting, for example, at T${}_{w}$ = 1.5 ps, all the off-diagonal observed peaks in previous snapshots gain additional strength. The off-diagonal peak (“F”), which originated due to the vibrational coupling among the C-H (2950 cm${}^{-1}$) and C=O in 2-Pyrrolidinone monomer, appeared in this snapshot. Longer waiting until 3 ps does not bring any further spectral features. Neither the shape nor the intensity of the peaks appeared until 1.5 ps, and did not change anymore. Therefore, it is fair enough to draw the conclusion that additional waiting would not bring any more information about the C-H and amide **I** coupling. Although different off-diagonal peaks appear in the spectra at different waiting times, in general, peak strength gradually increases with the waiting time and finally reaches saturation. The reason for the growth of the coupling strength is the chemical exchange. There exist two possible configurations of 2-Pyrrolidinone, for example, axial and equatorial [39] (see Figure 13b). Applying this to the first excitation pulse, the molecule goes from one configuration to the other, and finally back to the original. Getting back to the initial configuration takes some time, and as a result, the coupling strength of the C-H and amide **I** vibrations also grow slowly with time. In the saturation, different coupling peaks have different spectral strengths. This indicates that the coupling constant of amide **I** and different C-H bands are different. This is quite obvious, as different C-H bands are situated at different distances from the amide **I** bond.

To demonstrate the observation of molecular dynamics by 2DIR spectroscopy, only the time-dependent CHCO coupling is presented in this review article. However, 2DIR spectroscopy is not only restricted to this molecular motion, but is also able to observe the chemical exchange process like ultrafast carbon–carbon single-bond rotational motions in ethane molecules [20,77]. Many other chemical exchange processes have been observed by 2DIR spectroscopy and reported [128]. The making and breaking of hydrogen bonds were studied by 2DIR spectroscopy and already proved its potential to study ultrafast processes in biological molecules [104,110,126,138,139,140]. The amide bond dynamics have been reported on in many articles [24,107]. All this experimental evidence establishes the efficiency of 2DIR spectroscopy as an essential tool to study the molecular structure and their ultrafast dynamics. In particular, the ability to decouple the near-degenerate vibrations makes the 2DIR spectroscopy more suitable over other experimental methods for protein analysis.

## 7. Conclusions

In this review article, the applicability of two-dimensional vibrational spectroscopy to reveal the protein structure and dynamics have been presented. Vibrational spectroscopy was used as an essential experimental tool to study protein structure and dynamics for several decades. Conventional one-dimensional vibrational spectroscopy provides a one-dimensional projection of the available protein information onto a single frequency axis. On the contrary, 2DIR spectroscopy provides a two-dimensional projection of the relevant molecular information, and as a result, coupling constants, time-dependent structural information, and so forth are readily available from 2DIR spectroscopy. These information allow a deeper understanding of the protein structure and dynamics. This article reviews our theoretical and experimental efforts toward the understanding of the protein structure and its ultrafast dynamics. Using the model molecule, it was established here with several examples on how important it is to include coupling potentials in calculations or simultaneously exciting several bands to experimentally extract the structural information.

## Figures and Tables

**Table 1:** The harmonic and anharmonic C-H stretch vibrational frequencies are calculated using different level of computational methods and their assignment for deuterated MB. one-dimensional potential energy surfaces are calculated with the DF-MP2/aug-cc-pVTZ and pair coupling potential surfaces are calculated with DF-MP2/cc-pVDZ. Mode numbers are based on normal mode frequencies.

Mode	Frequency in cm${}^{-1}$				Assignment ^*a*^
	Harmonic	Diagonal	Experiment	Pair Coupling	
41	3092	3059	2998	2989	methyl C-H ss
42	3183	3255	2543	2550	methyl C-H as
43	3205	3208	2905	2889	phenyl C-H as
44	2341	2338	2185	2189	phenyl C-D as
45	3217	3213	2845	2832	methyl C-H ss
46	3222	3233	2954	2957	phenyl C-H as
47	3226	3191	3064	3065	phenyl C-H as
48	3238	3161	3022	3017	phenyl C-H as

^*a*^ as = asymmetric stretch, methyl C-H = Methyl group C-H bond, phenyl C-H = phenyl ring C-H bond, ss = symmetric stretch.
